# Epigenetic modifications in vascular inflammation

**DOI:** 10.3389/fimmu.2025.1711579

**Published:** 2025-12-04

**Authors:** Linyi Tang, Liyun Li, Yang Liu, Xin Zhang, Xinglin Wang, Zaiyong Zheng, Ping Yang, Haoran Wu

**Affiliations:** 1 Department of Cardiology,Guangyuan Hospital of Traditional Chinese Medicine, Guangyuan, China; 2 Key Laboratory of Medical Electrophysiology, Laboratory of Nucleic Acids in Medicine for National High-Level Talents, Ministry of Education and Medical Electrophysiological Key Laboratory of Sichuan Province, Institute of Cardiovascular Research, Southwest Medical University, Luzhou, Sichuan, China; 3 Department of Cardiology, The Affiliated Hospital of Southwest Medical University, Key Laboratory of Medical Electrophysiology, Ministry of Education, Institute of Cardiovascular Research, Basic Medicine Research Innovation Center for Cardiometabolic Diseases, Ministry of Education, Nucleic Acid Medicine of Luzhou Key Laboratory, Southwest Medical University, Luzhou, Sichuan, China; 4 Department of Cardiology, Panzhihua Central Hospital, Panzhihua, China

**Keywords:** vascular inflammation, epigenetics, DNA methylation, histone modification, RNA modification, non-coding RNA

## Abstract

Vascular inflammation is closely associated with the onset and progression of diseases, playing a pivotal role in the development of numerous acute and chronic conditions. The pathophysiological processes underlying vascular inflammation are highly complex, involving intricate cellular and molecular interactions. Recent scientific research suggests that epigenetics plays a significant role in vascular inflammation, offering new perspectives for deciphering its molecular mechanisms. Therefore, we review the role of epigenetics in vascular inflammation, explore its underlying mechanisms, and provide new insights for future clinical treatments.

## Graphical Abstract

**Figure:**
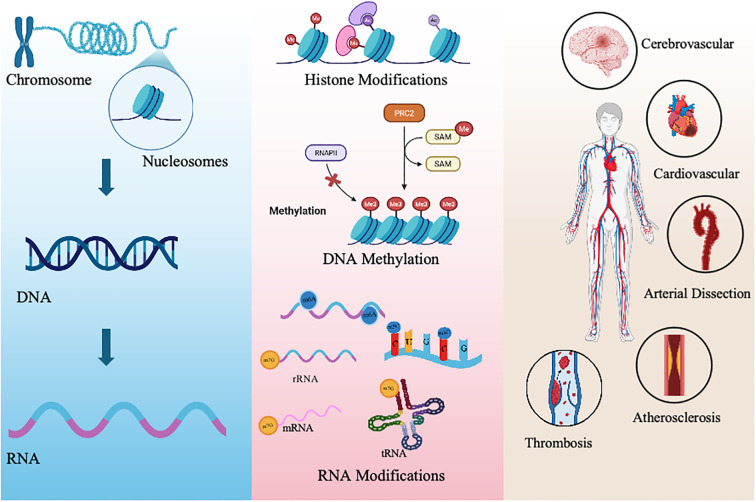
Some graphical elements in the Graphical Abstract were adapted from images provided by BioRender (https://www.biorender.com/).

## 1 Introduction

Vascular inflammation is a general term for a class of diseases where vascular injury results from an inflammatory response triggered by vascular irritation. Vascular inflammation contributes to various acute and chronic conditions, such as acute myocardial infarction, aneurysms, and atherosclerosis (1).Multiple pathological mechanisms contribute to the development of vascular inflammation. For instance, anti-neutrophil cytoplasmic antibodies specific for proteinase 3 (PR3-ANCA) and myeloperoxidase (MPO-ANCA) can induce neutrophil activation, leading to ANCA-associated vasculitis (2). Vascular endothelial cells (VECs) activated by specific cytokines or pro-inflammatory factors can secrete inflammatory mediators, triggering innate and adaptive immune responses that result in vascular inflammation (3); Henoch–Schönlein purpura (HSP) and IgA vasculitis arise from abnormal deposition of IgA immune complexes on small vessel walls during circulation, activating the complement system (particularly complement C3) and subsequently initiating a cascade of inflammatory reactions.

Except for capillaries, which are primarily composed of endothelium, blood vessels typically feature a three-layered structure: the intima, media, and adventitia. The intima, formed by vascular endothelial cells, serves as the first barrier of the vessel wall. Under physiological conditions, endothelial cells regulate vascular tone, smooth muscle cell proliferation, and immune cell adhesion to maintain vascular homeostasis (4). However, under certain risk factors, endothelial dysfunction occurs, disrupting vascular homeostasis and leading to abnormalities in vasoregulation, anticoagulation, anti-inflammation, and barrier function. Examples include increased secretion of adhesion molecules and chemokines, promoting leukocyte adhesion and migration; heightened permeability, facilitating inflammatory factor deposition in the vessel wall and exacerbating inflammatory responses. The media layer is primarily composed of vascular smooth muscle cells (VSMCs), which are crucial cells for maintaining vascular structure and function. Under inflammatory stimulation, VSMCs undergo phenotypic transformation and abnormal proliferation, secreting large amounts of cytokines, growth factors, and other products that further amplify the inflammatory response. Simultaneously, under inflammatory conditions, co-culture of VSMCs with human coronary artery endothelial cells (HCAECs) revealed that VSMCs amplify the expression of inflammation-related signaling pathway genes such as JAK/STAT, Jun, and NF-κB in HCAECs. Furthermore, inflammation-related transcription factors including NFKB1, RELB, IRF1, and STATs (1–6) exhibited more pronounced upregulation in the presence of VSMCs (5). Therefore, VSMCs significantly enhance the interaction between immune cells and endothelial cells by regulating endothelial gene expression and secretory functions, thereby further promoting the progression of vascular inflammation.

The adventitia, as the outermost layer of the vascular wall, contains loose connective tissue and fibroblasts. The connective tissue consists of numerous collagen fibers that anchor the vessel in place, while fibroblasts participate in fibrous proliferation during vascular injury repair. The adventitia is enveloped by perivascular adipose tissue (PVAT), which primarily consists of adipocytes, macrophages, and other infiltrating immune cells. During the inflammatory response associated with atherosclerosis, PVAT secretes cytokines that promote the development of inflammatory reactions (6). Macrophages, as a vital component of the immune system, are among the most plastic cell types within it. When activated, they can exhibit distinct states of polarization (7). Classically activated macrophages (M1) and alternatively activated macrophages (M2) represent the two primary subpopulations of macrophage polarization, exhibiting distinct functional characteristics (8). M1 macrophages secrete multiple pro-inflammatory cytokines that not only directly exacerbate vascular wall inflammation but also stimulate endothelial cells to express increased levels of adhesion molecules and chemokines. They further induce phenotypic transformation and proliferation in vascular smooth muscle cells (9). M2 macrophages primarily secrete anti-inflammatory factors, suppressing inflammation and promoting tissue repair (10). These two phenotypes can dynamically switch during different stages of vascular inflammation, finely regulating its progression. Therefore, understanding the regulatory mechanisms of vascular inflammation is crucial for disease monitoring and treatment.

Epigenetics refers to the phenomenon where gene expression and phenotype undergo developmental or environmentally induced changes without altering the DNA base sequence, yet these changes are heritable (11). Development or environment influences the expression of genetic information within DNA through mechanisms such as DNA methylation, histone modifications, non-coding RNAs, and RNA modifications, leading to tissue- or environment-specific expression patterns that produce heritable phenotypes. This process also underpins the diverse expression patterns of genes across different cell types. Epigenetics, as a key mechanism capable of dynamically regulating gene expression, plays a crucial role in the development and treatment of vascular inflammation. For instance, histone modifications at the MPO and PRTN3 gene loci may contribute to the pathogenesis of ANCA-associated vasculitis; DNA methylation levels at the PRTN3 promoter can also predict remission and recurrence in ANCA-associated vasculitis (2).

However, the specific mechanisms of epigenetics in vasculitis remain unclear at present, hindering the advancement of vasculitis treatment. Therefore, this paper comprehensively reviews research on epigenetics and vasculitis, explores the mechanisms of various epigenetic alterations in vasculitis, and discusses the application prospects of epigenetics-based therapies for vasculitis.

## 2 Epigenetic mechanisms in vascular inflammation

Vascular inflammation is intrinsically linked to the progression of cardiovascular disease. Research indicates that vascular inflammation is a key factor in the early stages of atherosclerosis (12). During the inflammatory process in blood vessels, endothelial cells, macrophages, and smooth muscle cells undergo morphological, functional, and metabolic changes in response to inflammatory stimuli. Vascular inflammation also serves as a critical mechanism in the development, progression, and rupture of abdominal aortic aneurysms (AAA).

### 2.1 Vasculitis and DNA methylation

DNA methylation is an epigenetic modification process catalyzed by DNA methyltransferases (DNMTs), wherein a methyl (-CH_3_) group is covalently attached to the 5th carbon atom of the cytosine (C) base. The DNMT3 and DNMT1 families are responsible for establishing and maintaining methylation, respectively, a process that does not alter the DNA base sequence. DNA methylation plays crucial roles in mammalian development, including regulating gene expression and maintaining genomic stability (13) (**Table 1**).

**Table 1:** Summary of the regulatory mechanisms of vasculitis by DNA methylation and histone modifications.

Enzyme	Type	Epigenetic mechanism	Actions on vasculitis	Reference
TET2	DNA methylation	Converting 5-methylcytosine (5mC) to 5-hydroxymethylcytosine (5hmC)	Reduce VSMC apoptosis and endothelium thickening	(14, 15)
UHRF1	DNA methylation	When activated, it induces hypermethylation of CpG sites in the promoter region of miR-26b-3p, thereby suppressing the transcriptional expression of downstream targets.	VSMC Phenotype Conversion	(17)
JMJD3	Histone Modification - Methylation	Specific removal of histone H3 lysine 27 trimethylation (H3K27me3) and dimethylation (H3K27me2) modifications	Macrophage Phenotypic Differentiation	(29–31)
DOT1L	Histone Modification - Methylation	Directly modulates the methylation level of H3K79 on the genomic region of Nf-κb genes	Promotes VSMC proliferation and migration while inhibiting VSMC differentiation	(34)
MOF	Histone Modification - Acetylation	Acetylation of UHRF1 at the K670 site enhances its E3 ubiquitin ligase activity, thereby promoting histone H3 ubiquitination	Regulating Phenotypic Conversion of VSMCs	(18, 37)

The TET (ten-eleven translocation) protein family comprises a group of DNA demethylases capable of regulating various epigenetic responses by converting 5-methylcytosine (5mC) into 5-hydroxymethylcytosine (5hmC) (14). This process constitutes one of the essential stages of DNA demethylation. As a member of the TET protein family, TET2 reduces VSMC apoptosis and intimal thickening under interferon stimulation (15). The phenotypic conversion of vascular smooth muscle cells is not a singular, isolated process; it is directly influenced by multiple epigenetic mechanisms and also affected by crosstalk between vascular endothelial cells. Activation of the CD137 signaling pathway in endothelial cells attenuates their protective effect against VSMCs, suggesting that the CD137-TET2 signaling pathway offers a novel therapeutic target for preventing and treating excessive VSMC proliferation or restenosis in aortic stenosis (AS) (16).

Upon activation, the epigenetic regulator UHRF1 (ubiquitin-like containing PHD and RING finger domains 1) induces hypermethylation of CpG sites in the promoter region of miR-26b-3p, directly suppressing the transcriptional expression of its downstream target miR-26b-3p.This regulates matrix metalloproteinase (MMP) activity and vascular smooth muscle cell (VSMC) phenotypic conversion (17). Studies indicate that in proliferating VSMCs, UHRF1 binds to the promoter regions of cell cycle inhibitor genes. Through DNA methylation and histone modifications, it suppresses their expression, thereby releasing cell cycle arrest and promoting vascular smooth muscle cell proliferation (18). Concurrently, UHRF1 binds to the promoters of key pro-differentiation genes in VSMCs, suppressing their expression through similar epigenetic modifications. This induces a shift from a contractile phenotype (differentiated state) to a synthetic phenotype (dedifferentiated state) (18), thereby exacerbating inflammatory responses.

Although UHRF1 lacks intrinsic enzymatic activity, it binds to hypomethylated DNA through its SRA domain, recruits DNA methyltransferase 1 (DNMT1) to the site to maintain DNA methylation, and simultaneously interacts with histone deacetylase 1 (HDAC1) to participate in histone deacetylation, jointly suppressing target gene expression.

In Kawasaki Disease (KD), an acute self-limiting vasculitis of unknown etiology, DNA methylation modulates gene expression by modifying CpG sites (primarily in promoter regions) of specific genes. For instance, Toll-like receptors (TLRs) such as TLR1, 2, 4, 6, 8, and 9, exhibiting hypomethylation in their promoter regions, leading to mRNA upregulation and mediating inflammatory responses (19); The S100A gene family exhibits hypomethylation during the acute phase of KD and hypermethylation during recovery, dynamically regulating leukocyte transendothelial migration (20); Among NOD-like receptors (NLRs), hypomethylation of the NLRC4 gene upregulates its expression, promoting caspase-1 activation and IL-1β synthesis, while hypermethylation of the NLRP12 gene reduces its expression. Together, these effects exacerbate the inflammatory response (21).

The functions of key molecules such as DNMTs, the TET protein family, and UHRF1 influence vascular smooth muscle cell proliferation, phenotypic switching, and inflammatory responses by regulating gene methylation states. These mechanisms play roles in vascular diseases like atherosclerosis and Kawasaki disease, offering potential therapeutic targets for disease treatment.

### 2.2 Vasculitis and histone modifications

Under normal physiological conditions, histone modifications are crucial for ensuring DNA replication fidelity and damage repair. They participate in DNA replication and repair processes, safeguarding genomic stability. Additionally, they regulate key cell cycle genes and differentiation-related genes, ensuring normal cell proliferation and differentiation while preventing abnormal proliferation or differentiation disorders. Histone modifications also participate in specialized physiological processes such as germ cell development and metabolic regulation, fulfilling specific cellular functional requirements (22). The N-terminal tails of histone core proteins undergo various covalent modifications including methylation, acetylation, and phosphorylation (23). Among these, histone methylation occurs on arginine and lysine residues, catalyzed by two classes of proteins: the protein arginine methyltransferase family and the SET-domain-containing methyltransferase family (24). Histone modifications have emerged as critical regulators of chromatin dynamics and gene activity.

Hellenthal and colleagues demonstrated in their research on abdominal aortic aneurysms (AAA) that inflammation is a key characteristic of AAA pathogenesis, with inflammatory cells extensively infiltrating the vascular wall in both the intima and adventitia (25). Monocytes/macrophages are highly significant inflammatory cells. Macrophage infiltration drives pathological vascular remodeling. During this remodeling process, monocytes—as the primary myeloid cell population involved in the inflammatory process—are recruited into aortic tissue, where they differentiate into macrophages. These differentiated cells then collaborate with specialized tissue-resident macrophages (26). Epigenetic modifications regulate the expression of immune mediators in macrophages both *in vivo* and *in vitro (*27). The histone demethylase JMJD3, containing a Jumonji (JmjC) domain, belongs to the Fe²^+^- and α-ketoglutarate (2OG)-dependent dioxygenase superfamily (28). It specifically removes trimethylation (H3K27me3) and dimethylation (H3K27me2) modifications from histone H3 lysine 27, thereby regulating gene expression (29) (30). The epigenetic enzyme JMJD3 plays a critical role in macrophage phenotypic differentiation. Research by Satoh et al. confirmed that JMJD3 influences macrophage polarization, and JMJD3 inhibitors reduce proinflammatory factor levels in LPS-stimulated macrophages (31). Following tissue single-cell sequencing of human AAA models, Davis identified increased JMJD3 expression in aortic monocytes/macrophages, leading to upregulation of inflammatory immune responses. Conversely, targeted inhibition of JMJD3 significantly reduced AAA expansion and alleviated macrophage-mediated inflammation (32).

Histone H3 lysine residue 79 is located within the globular domain and is modified by Disruptor of telomeric silencing-1-like (DOT1L). DOT1L is a key enzyme catalyzing histone H3K79 methylation, belonging to the histone lysine methyltransferase family. This protein exhibits intrinsic H3K79-specific histone methyltransferase (HMTase) activity both *in vitro* and *in vivo (*33). DOT1L promotes vascular smooth muscle cell (VSMC) proliferation and migration while inhibiting VSMC differentiation. In inflammation regulation, it directly modulates the methylation level of H3K79 on the genomic region of NF-κB genes. Following NF-κB1/2 activation, DOT1L further induces VSMC secretion of chemokines such as CCL5 (C-C chemokine ligand 5) and CXCL10 (C-X-C chemokine ligand 10), promoting immune cell recruitment to the vascular wall and exacerbating local inflammation (34). In macrophages, DOT1L indirectly suppresses excessive activation by maintaining lipid synthesis homeostasis (particularly via the SREBP pathway). DOT1L deficiency or inhibition induces a lipid synthesis defect-enhanced inflammation phenotype, accelerating chronic inflammatory disease progression (35). Inhibiting DOT1L activity may improve atherosclerotic disease prognosis by reducing inflammation and stabilizing plaques. This mechanism reveals that epigenetic modifications (H3K79 methylation) influence macrophage function through metabolic-inflammatory cross-regulation.

MOF (males absent on the first) is a histone acetyltransferase primarily responsible for acetylating lysine 16 of histone H4 (H4K16) (36). MOF acetylates the K670 site of UHRF1, while HDAC1 (histone deacetylase 1) deacetylates this site. This acetylation modification enhances UHRF1’s E3 ubiquitin ligase activity, thereby promoting histone H3 ubiquitination. This process is crucial for recruiting DNMT1 (DNA methyltransferase 1) and maintaining DNA methylation (37). Furthermore, UHRF1 promotes H3K27me3 modification in target gene promoter regions, synergistically inhibiting gene expression with DNA methylation to regulate the phenotypic transformation of VSMCs (18).

Histone modifications (including methylation, acetylation, etc.) are essential for normal physiological processes and also regulate chromatin dynamics and gene activity. In vascular diseases such as abdominal aortic aneurysms, histone modification-related enzymes including JMJD3, DOT1L, and MOF participate in inflammatory responses and vascular remodeling by regulating macrophage polarization, inflammatory cytokine expression, and vascular smooth muscle cell function. Targeting their inhibitors or modulating their activity may offer new directions for disease intervention.

### 2.3 Vasculitis and non-coding RNA

Non-coding RNA (ncRNA) represents a class of RNA molecules that are widely present in the human transcriptome yet exhibit extremely low expression levels, complex structures, and potential regulatory functions. While ncRNA does not encode proteins, it can regulate gene expression through three core mechanisms: transcriptional regulation, post-transcriptional regulation, and epigenetic regulation (38). These molecules play crucial roles in cellular differentiation, development, and disease onset (39).

Non-coding RNAs can be categorized by size into small non-coding RNAs (ncRNAs) and long non-coding RNAs (lncRNAs). Small ncRNAs include microRNAs (miRNAs), tRNA-derived small RNAs (tsRNAs), and PIWI-interacting RNAs (piRNAs). Among these, miRNAs have been most extensively studied; they are approximately 22nt in length and regulate gene expression by binding to target mRNAs. lncRNA exceeds 200nt in length and encompasses pseudogenes and circular RNA (circRNA). While lncRNA exhibits low expression levels and poor evolutionary conservation, it is abundant and diverse in function. circRNA forms through reverse splicing, demonstrates high stability, and is widely expressed in cancer (38).

Non-coding RNAs can target inflammation-related molecules and exert bidirectional regulatory effects—both anti-inflammatory and pro-inflammatory—in vascular inflammation, making them an active area of current research (**Table 2**).

**Table 2:** Summary of non-coding RNA regulation mechanisms in vasculitis.

nc RNA	Type	Epigenetic mechanism	Actions on vasculitis	Reference
miR-126	miRNA	Targeting molecules such as SPRED1 and PI3KR2 activates the PI3K/AKT signaling pathway and enhances the VEGF signaling pathway	Promoting endothelial cell survival and angiogenesis	(41, 42)
miR-92a	miRNA	Targeting genes such as Krüppel-like factor 2 (KLF2), KLF4, and sirtuin 1 (SIRT1)	Endothelial dysfunction	(43)
miR-92a	miRNA	Mediating communication between vascular endothelial cells and vascular smooth muscle cells	VSMC Phenotypic Conversion	(43)
miR-145	miRNA	Targeted inhibition of UHRF1 translation downregulates UHRF1	Inhibits VSMC proliferation and promotes their differentiation toward a contractile phenotype	(18)
MALAT1	lncRNA	Interaction with NF-κB reduces the production of cellular inflammatory factors	Reduce the expression of TNF-α and IL-6 in macrophages	(50)
MAP3K4	lncRNA	Regulates inflammation through cis-regulation of the p38 MAPK pathway	Reduce monocyte adhesion to endothelial cells and decrease the expression of TNF-α, IL-1β, and COX2 in macrophages	(51)
circHIPK3	circRNA	Increase levels of inflammatory cytokines	Promotes abnormal proliferation of vascular endothelial cells and vascular leakage	(55)
circ-Sirt1	circRNA	Inhibiting NF-κB p65 nuclear translocation and promoting SIRT1 expression to suppress nuclear NF-κB activity	Reduce vascular inflammatory responses and neointimal hyperplasia, and inhibit the phenotypic transformation of VSMCs	(56)

#### 2.3.1 miRNA

Approximately 150 types of microRNAs in the human genome are associated with the cardiovascular system, with 30–35 of them validated through *in vivo* experiments to play crucial roles, providing potential targets for targeted therapy (40).

miRNAs play a crucial role in the proliferation, migration, and angiogenesis of vascular endothelial cells. For example, miR-126 activates the PI3K/AKT signaling pathway by targeting molecules such as SPRED1 and PI3KR2, thereby enhancing the VEGF signaling pathway to promote endothelial cell survival and angiogenesis. When its levels decrease, Spred1 and PI3KR2 overexpression inhibit the MAPK and PI3K signaling pathways, disrupting vascular factor signaling and impairing vascular repair capacity (41), which exacerbates atherosclerosis (42). Elevated miR-92a levels can cause endothelial dysfunction by targeting genes such as Krüppel-like factor 2 (KLF2), KLF4, and sirtuin 1 (SIRT1). Furthermore, miR-92a promotes vascular smooth muscle cell (VSMC) phenotypic conversion by mediating communication between vascular endothelial cells and VSMCs, and its antagonists may serve as potential therapeutic agents (43).

Among various microRNAs, both miR-92a and miRNA-145 play crucial roles in the differentiation process of vascular smooth muscle cells (VSMCs) (44), though their specific mechanisms differ slightly. Although miR-145 belongs to post-transcriptional regulatory factors, it indirectly participates in epigenetic regulation by modulating UHRF1 expression, serving as a crucial upstream regulator of VSMC phenotypic switching. The specific mechanism involves miR-145 overexpression targeting UHRF1 translation to downregulate its expression, thereby inhibiting VSMC proliferation and promoting their differentiation toward a contractile phenotype. Conversely, reduced miR-145 expression weakens its inhibition of UHRF1, leading to UHRF1 accumulation that promotes VSMC proliferation and dedifferentiation (18).Moreover, miRNA-145 and miR-143 act synergistically to regulate the quiescence and proliferation phenotypes of smooth muscle cells (45).

miRNAs also function as protective epigenetic mechanisms in vascular inflammation. For instance, miR-223 reduces leukocyte adhesion by regulating inflammatory responses in vascular endothelial cells. Furthermore, the Ripk3/C/EBPβ/miR-223-3p pathway forms a negative feedback regulatory network governing foam macrophage programmed necrosis, thereby slowing atherosclerosis progression (46); miR-100, an endothelium-enriched microRNA, exerts anti-inflammatory effects by promoting autophagy and inhibiting pro-inflammatory signaling and endothelial cell activation through autophagy-dependent pathways (47). Research indicates that miR-100-5p effectively suppresses mTOR and its downstream signaling pathways, exerting significant effects on inflammatory responses both *in vivo* and *in vitro (*48). As miR-100-5p is a low-expression miRNA, further studies are needed to determine whether it serves as a primary factor in inflammatory diseases.

#### 2.3.2 lncRNA

Certain non-coding sequences within the human genome are transcribed into long non-coding RNAs (lncRNAs). As an emerging regulatory factor gaining significant attention in recent years, lncRNAs play a crucial role in the progression of numerous diseases, including cardiovascular disease (CVD). Vascular inflammation, a critical process in the development of cardiovascular disease, may involve lncRNAs expressed with cell- and tissue-specific patterns. This suggests lncRNAs hold substantial potential as novel biomarkers for disease treatment (49).

MALAT1 is a highly conserved long non-coding RNA initially identified as associated with lung adenocarcinoma metastasis. Subsequent studies revealed its critical role in vascular inflammation. MALAT1 regulates inflammatory responses in a cell-specific manner. It interacts with NF-κB to reduce cellular cytokine production. Macrophages exhibit MALAT1 upregulation following lipopolysaccharide (LPS) stimulation, while knocking down MALAT1 increases LPS-induced TNF-α and IL-6 expression (50).

lncRNA-MAP3K4 has also been demonstrated to be a crucial inflammatory regulator. During the inflammatory process, lncRNA-MAP3K4 accumulates in the vascular wall and modulates inflammation through cis-regulation via the p38 MAPK pathway. Studies indicate that knocking down lncRNA-MAP3K4 reduces the expression of key inflammatory mediators (such as ICAM-1 and MCP-1) in vascular endothelial cells or smooth muscle cells, decreases monocyte adhesion to the endothelium, and lowers the expression of TNF-α, IL-1β, and COX2 in macrophages (51).

lncRNA SMILR, a long non-coding RNA involved in vascular smooth muscle cell proliferation, is highly expressed in atherosclerosis (52), particularly activated in unstable plaques (53). This suggests that lncRNA SMILR may contribute to the formation and progression of vascular inflammation by promoting vascular smooth muscle cell proliferation and migration. lncRNA-Cox2 modulates macrophage polarization. Overexpression of lncRNA-Cox2 enhances the effects of LPS on inflammation and macrophage polarization, whereas knockdown of lncRNA-Cox2 attenuates macrophage polarization and inflammatory responses (54). Current studies demonstrate the regulatory role of lncRNAs in inflammation, though the specific mechanisms require further elucidation.

#### 2.3.3 circRNA

Circular non-coding RNAs are a class of endogenous non-coding RNAs featuring covalently closed circular structures. They exhibit high structural stability, diverse functions, and tissue-specificity and developmental stage-specificity, with significant expression differences across different cells or tissues. Different circular RNAs exhibit distinct patterns in vascular inflammation. Studies indicate that circular HIPK3 (circHIPK3) participates in diabetic retinal vascular pathology through the circHIPK3-miR-30a-3p-VEGFC/FZD4/WNT2 axis. High expression of circHIPK3 not only promotes abnormal proliferation of vascular endothelial cells and vascular leakage but also exacerbates vascular inflammation by upregulating inflammatory factor levels (55). Meanwhile, circ-Sirt1—a circular RNA derived from the SIRT1 host gene—mitigates vascular inflammatory responses and neointimal hyperplasia through a dual mechanism: suppressing NF-κB nuclear translocation and promoting SIRT1 expression to inhibit nuclear NF-κB activity. It also synergistically suppresses the inflammatory phenotypic switch in VSMCs, exerting protective effects in vascular diseases such as atherosclerosis (56).

Non-coding RNAs (including miRNA, lncRNA, circRNA, etc.) do not encode proteins but participate in gene expression through multiple regulatory mechanisms, exerting both anti-inflammatory and pro-inflammatory effects in vascular inflammation. Among these, miRNAs (such as miR-126, miR-92a) regulate the function of vascular endothelial cells and smooth muscle cells, representing potential therapeutic targets for cardiovascular diseases; lncRNAs (e.g., MALAT1, SMILR) exhibit cell- and tissue-specificity, regulating inflammatory cytokine expression in vascular inflammation and demonstrating potential as biomarkers; circRNAs (e.g., circHIPK3, circ-Sirt1) possess structural stability and participate in vascular inflammation regulation through specific molecular pathways, exhibiting differential effects.

### 2.4 Vasculitis and RNA modification

RNA modifications represent a crucial form of epigenetics, belonging to the fields of epigenomics and epigenomics. Together, these domains regulate gene expression in eukaryotic organisms. RNA modifications constitute a dynamic post-transcriptional chemical modification process. By adding, removing, or altering specific chemical groups—such as methyl, acetyl, or uridine—they regulate the fate and function of RNA (57), thereby influencing multiple stages of gene expression. The dynamic nature of RNA modifications is reflected in a write-read-erase regulatory network composed of three core protein categories (or complexes) (57). This mechanism fundamentally expands genomic information diversity by altering RNA’s chemical structure, rather than modifying its nucleotide sequence (58).

RNA methylation is the most common epigenetic modification of RNA nucleotides, primarily encompassing distinct modifications such as N6-methyladenosine (m6A), 5-methylcytosine (m5C), 7-methylguanosine (m7G), and N1-methyladenosine (m1A) (**Table 3**).

**Table 3:** Summary of RNA modifications in the regulatory mechanisms of vasculitis.

Enzyme/RNA	Type	Epigenetic mechanism	Actions on vasculitis	Reference
METTL3	m6A -methyltransferase	Targeted regulation of NLRP3 and KLF4 expression activates the NF-κB pathway and promotes monocyte adhesion	Exacerbate the inflammatory response in endothelial cells	(60)
FTO	m6A-demethylase	Specific removal of m6A methylation from TNIP1 mRNA activates the NF-κB pathway to promote the release of inflammatory mediators	Causes vascular endothelial dysfunction	(61)
NSUN2	m5C-Methyl Transferase	Promotes the translation of ICAM-1 mRNA through methylation	Enhanced adhesion between leukocytes and endothelial cells	(67)
METTL1	m7G-methyltransferase	Methylation-dependent promotion of VEGFA mRNA translation	Promote angiogenesis	(70)
GlycoRNA	RNA glycosylation	Modifying RNA with acp3U as the N-glycan binding site	Enhance the interaction between macrophages and vascular endothelial cells	(73, 74)

#### 2.4.1 m6A modification

m6A modification is one of the most common epigenetic modifications of the transcriptome and represents a reversible chemical modification. A protein complex comprising methyltransferases, demethylases, and binding proteins enables the addition, removal, and recognition of m6A-modified sites—specifically, the covalent addition of a methyl group to the nitrogen-6 position of adenosine—dynamically regulating RNA metabolism and function (59).

METTL3, a type of methyltransferase, can specifically regulate the expression of NLRP3 and KLF4 in endothelial cells responding to oscillatory stress. This activates the NF-κB pathway and promotes monocyte adhesion, ultimately exacerbating the inflammatory response in endothelial cells (60).

FTO is a demethylase that specifically removes m6A methylation from TNIP1 mRNA, thereby reducing its stability and suppressing its expression. This activates the NF-κB pathway, promoting the release of inflammatory mediators and leading to vascular endothelial dysfunction such as leakage and capillary abnormalities. Knocking down FTO expression in endothelial cells (ECs) reduces inflammation (61).

YTHDF3 belongs to the DF family and functions as an m6A reader involved in RNA metabolism. It specifically recognizes m6A modifications to regulate mRNA maturation, translation, and degradation, playing a crucial role in the initial stages of translation (62). Similarly, m1A can promote macrophage polarization in aortic inflammation through YTHDF3, thereby influencing target gene expression and affecting AAA progression (63).

#### 2.4.2 m5C modification

m5C modification refers to the process where the cytosine base undergoes methylation at the 5th carbon atom to form 5-methylcytosine. This modification plays a central role in multiple RNA metabolic processes, including mRNA output, RNA stability, and translation (64). Methyl transferases (writers) and demethylases (erasers) reversibly regulate m5C methylation levels, while most m5C functions are mediated through binding to specific proteins (readers) (65).

NSUN2 is a member of the NOP2/SUN family, an RNA m5C methyltransferase present in eukaryotes. NSUN2 catalyzes 5mC methylation at positions C48/49/50, which contributes to maintaining tRNA stability and mediates methylation in the tRNA variable loop to facilitate protein synthesis (66). Research indicates that NSUN2 promotes ICAM-1 mRNA translation through methylation, enhancing adhesion between leukocytes and endothelial cells. Pro-inflammatory factors such as TNF-α or homocysteine activate NSUN2 via Aurora-B (a protein kinase), exacerbating inflammatory responses. Conversely, NSUN2 deficiency exerts a protective effect against vascular inflammation (67).

#### 2.4.3 m7G modification

m7G modification is a post-transcriptional base modification commonly occurring at the 5’ end of tRNA, rRNA, and eukaryotic mRNA (68). m7G modification plays a crucial role in RNA processing, stability, maturation, and translation, making it an indispensable modification process in RNA metabolism. Methyl transferase-like 1 (METTL1) is the methyltransferase responsible for m7G modification, dynamically regulating its levels (69).

METTL1 promotes angiogenesis by enhancing VEGFA mRNA translation in an m7G methylation-dependent manner (70). Although the precise mechanism of m7G modification in AAA remains incompletely elucidated, m7G-associated genes (CYFIP1, EIF3D, EIF4E3, NSUN2, NUDT11) show positive correlations with AAA-related scores (71). It can be inferred that m7G modification may also exert certain effects on the progression of vascular inflammation. Future studies should validate its specific role in the development and progression of vascular inflammation through further experimental investigations.

#### 2.4.4 GlycoRNA

Glycosylation is one of the most prevalent and crucial modifications of biomolecules. Previously, RNA was not considered a primary target for glycosylation, which was primarily observed in proteins and lipids. However, recent studies have revealed that RNA serves as the third major glycosylation carrier alongside proteins and lipids.

In mammalian cells, a class of small non-coding RNAs known as glycoRNAs exists on the cell surface, bearing N-glycosylation modifications (72). Unlike traditional RNA modifications such as m6A and m5C, which are concentrated internally, RNA glycosylation occurs at the cell surface. GlycoRNA consists of RNAs modified with secretory N-glycans. The modified RNA base 3-(3-amino-3-carboxypropyl)uridine (acp3U) serves as the attachment site for N-glycans in glycoRNA (73). Using ARPRA, a highly sensitive and selective method for direct visualization of glycoRNA, we discovered that glycoRNA increases during LPS-induced inflammatory responses and demonstrated that glycoRNA enhances interactions between macrophages and vascular endothelial cells (74). Further studies revealed that neutrophil glycoRNA is cell-autonomously produced, located on the extracellular surface, and recognized by P-selectin expressed by endothelial cells. Eliminating cell surface RNA significantly reduced neutrophil recruitment to inflammatory sites in mice and diminished neutrophil adhesion and migration toward endothelial cells (75).

RNA modifications represent a crucial form of epigenetic regulation, influencing gene expression through a write-read-erase regulatory network. Among these, RNA methylation (m6A, m5C, m7G, etc.) and the recently discovered GlycoRNA are closely associated with vasculitis. m6A-associated proteins (e.g., METTL3, FTO), the m5C methyltransferase NSUN2, and the m7G methyltransferase METTL1 participate in vasculitis by regulating inflammatory pathways, cell adhesion, or angiogenesis, respectively. GlycoRNA enhances interactions between immune cells and vascular endothelial cells, thereby promoting inflammatory responses.

## 3 Targeted epigenetic therapeutic strategies for vasculitis

Epigenetic modifications can influence the onset and progression of vascular inflammation, suggesting that regulating epigenetics may offer novel approaches and insights for treating vascular inflammatory diseases.

DNA methylation is catalyzed by DNA methyltransferases (DNMTs), and current therapeutic strategies targeting DNA methylation primarily focus on DNMT inhibitors. Drugs targeting DNMTs include cytosine analogs, oligonucleotide drugs, DNA binders, and S-adenosylmethionine competitors (76). Among these, oligonucleotide drugs prevent DNMTs from binding to specific gene promoters, thereby inhibiting DNA methylation; DNA binders target the cofactor binding sites of DNMTs; notably, cytosine analogs such as 5-Azacytidine (5-AzaC) and 5-Aza-CdR (trade name, decitabine) covalently bind to DNMTs, thereby inhibiting their activity. Studies have shown that 5-Aza-2’-deoxycytidine inhibits DNA methylation, thereby reducing macrophage inflammatory responses and achieving the goal of improving AS (77).

Therapeutic approaches targeting histone modifications focus on histone methylation and histone acetylation. The histone methylation inhibitor GSK 126 has been demonstrated to be a highly selective inhibitor of the histone N-methyltransferase EZH2. EZH2 participates in suppressing gene expression through methylation of histone H3 at H3K27. GSK 126 inhibits H3K27me3, thereby reducing the expression of pro-inflammatory genes (78). Histone acetyltransferase inhibitors (HATi) and histone deacetylase inhibitors (HDACi) are potential modulators of histone acetylation. HDACi comprise four major classes: cyclic peptides, aliphatic acids, and benzamides. They function by inhibiting histone deacetylation at target gene promoters, thereby reactivating silenced genes (79). Suberoylanilide hydroxamic acid (SAHA) is a type of HDAC inhibitor that exerts anti-inflammatory effects in endothelial cells via a Kruppel-like factor 2 (KLF2)-dependent mechanism (80); The specific HDAC inhibitor Trichostatin A (TSA) mediates anti-inflammatory properties to alleviate AS through the key mediator PPARγ (Peroxisome proliferator-activated receptor gamma) (81). Different HDAC classes possess distinct selective inhibitors. For instance, valproic acid (VPA), an I/IIa selective HDACi, reduces inflammatory responses in macrophages (82); TMP195, a selective inhibitor of Class IIa HDAC, achieves anti-inflammatory effects by decreasing endothelial activation and leukocyte recruitment within macrophages (83).

DNA methylation is frequently accompanied by histone deacetylation, making the combination of DNA methylation inhibitors with HDAC inhibitors a promising therapeutic approach for vascular inflammatory diseases.

Therapeutic approaches targeting m6A regulators focus on the writer enzyme METTL3 or the eraser enzyme FTO. For instance, STM2457 is a novel small-molecule inhibitor of METTL3, exhibiting high specificity for METTL3. It currently plays a significant role in tumors and holds promise for treating vascular inflammation (84); RSM3 is a novel peptide inhibitor of METTL3 that reduces METTL3 protein expression levels, leading to a marked decrease in cellular methylation levels (85).

Epigenetic editors are currently attracting significant attention. Recent research reports a biomimetic liposome loaded with fluid shear-responsive miR-10a that can target inflammatory macrophages in atherosclerotic plaques. The mechanism involves miR-10a restoring mitochondrial energy metabolism in M1 macrophages while significantly increasing histone acetylation levels (H3K9Ac), thereby reprogramming M1 macrophages to an M2 phenotype (anti-inflammatory phenotype) to alleviate disease progression (86).

More targeted epigenetic therapies will emerge in the future, and we look forward to more effective and specific treatment approaches.

Additionally, gene expression is influenced by various environmental factors such as nutrition, lifestyle, and exercise, all of which serve as important epigenetic regulatory factors (87). Nutrients in the diet can directly affect gene expression by regulating DNA methylation through the methyl donor AdoMet (88). Dietary polyphenols, including soy, genistein, and resveratrol, among others, exert their effects. Specifically, soy polyphenols can block DNA methyltransferases and histone deacetylases (89). Sulforaphane in broccoli can normalize DNA methylation and activate the expression of miR-140 (90). Thus, environmental factors represent a critical, often overlooked component in disease treatment and remission. Exercise, as an epigenetic modulator, also plays a key role in gene expression. Regular physical activity regulates methylation of genes related to muscle growth and metabolism, alters fat storage, and reduces the risk of associated diseases (91). Diet and lifestyle interventions can serve as alternative approaches for disease prevention and treatment, providing theoretical support for personalized health strategies.

## 4 Conclusions and prospective

In this review, we summarize how epigenetics precisely regulates inflammation-related gene expression in vascular endothelial cells, vascular smooth muscle cells, and macrophages through DNA methylation, histone modifications, non-coding RNAs, and RNA modifications, thereby controlling vascular inflammation. Key findings include: UHRF1-mediated hypermethylation in DNA methylation exacerbates VSMC phenotypic changes and inflammation; histone modifications regulate macrophage function to influence inflammation; non-coding RNAs exert bidirectional regulation on inflammation; RNA modifications via m6A, m5C, and m7G. VSMC phenotypic switching is jointly influenced by non-coding RNAs, DNA methylation, and histone modifications. These three epigenetic modifications connect VSMCs and macrophages in vascular inflammation through UHRF1, DOT1L, and JMJD3 link VSMCs and macrophages in vascular inflammation. Histone modifications function in both VSMCs and macrophages. Following DNA methylation and histone modifications, VSMCs can influence macrophages through chemokine secretion, suggesting that epigenetic regulation enables bidirectional interactions between different cell types.

This not only reveals the role of epigenetics in vascular inflammation under physiological and pathological conditions but also provides systematic insights and directions for its treatment. Vascular inflammation is closely associated with the onset and progression of various diseases. The novel perspective of utilizing epigenetics for targeted therapy against vascular inflammation also lays the foundation for developing novel anti-inflammatory treatment strategies for diseases such as atherosclerosis, aortic aneurysms, and Kawasaki disease. We focuses on exploring the primary mechanisms of epigenetic modifications and the relationship between their associated regulatory factors and the occurrence and progression of vascular inflammation. In the future, epigenetic regulatory factors may become a breakthrough point for the effective prevention and treatment of more diseases.

Numerous genes within the epigenome are also associated with vascular inflammation: the PTX3 gene plays a crucial regulatory role in inflammation (92); ANCA-associated vasculitis correlates with the HLA-DPB1 gene; giant cell arteritis correlates with the HLA-B52 gene; NR1H3 is a candidate gene regulating AAA formation … Epigenomic studies reveal that subclinical atherosclerosis mediated by systemic inflammation is highly correlated with accelerated epigenetic aging (93). This association underscores the importance of targeting inflammation to prevent cardiovascular disease.

Although research on epigenetic regulation of vascular inflammation is advancing rapidly—demonstrating its ability to influence the initiation, progression, and resolution of vascular inflammation through the establishment of cell-specific spatiotemporal dynamic regulatory networks—many unresolved questions remain. These include the cross-talk between epigenetic mechanisms and numerous other molecules in blood vessels, as well as the need to elucidate cell-specific mechanisms. Future research should integrate single-cell technologies, specific models, and other direct or indirect regulatory systems to deeply decipher epigenetic mechanisms. MicroRNAs (miRNAs) participate in the epigenetic regulation of inflammatory pathways, yet their expression in microvasculature and their relationship with chronic inflammation and metabolic dysfunction remain unclear. Furthermore, treatments targeting DNA methylation lack specificity, potentially leading to adverse reactions (94).It is noteworthy that DNA methylation is dynamically reversible. Designing specific drugs targeting DNA methylation markers for particular individuals and disease stages represents a new direction for future targeted epigenetic therapies. The emergence of epigenetic editing technologies offers a glimpse into the era of epigenetic therapeutics to come.

Research on cell surface RNA remains in its infancy, with RNA-mediated regulation of cellular functions representing an entirely new field. Previous studies have reported that Sidt gene knockout significantly reduces glycoRNA production in neutrophils (75). Future discoveries regarding other genes regulating cell surface RNA are anticipated, suggesting rapid progress in this area. GlycoRNA also holds promise as a future tool for epigenetic therapy. RNA-binding proteins (RBPs) form nanoclusters with glycoRNAs on the surface of living cells, regulating key signaling pathways and mediating the entry of trans-activator of transcription (TAT) into cells, providing specific binding sites for its action (95). This offers inspiration for designing future drug delivery systems, potentially enabling the creation of drugs that precisely target diseased cells.
